# Spontaneous breathing test conducted with and without automatic tube compensation does not differ from a metabolic standpoint

**DOI:** 10.1186/2197-425X-3-S1-A668

**Published:** 2015-10-01

**Authors:** AF Lago, EC Gonçalves, EC Silva, MG Menegueti, EA Nicolini, M Auxiliadora-Martins, AC Gastaldi, A Basile-Filho

**Affiliations:** Ribeirão Preto Medical School, University of São Paulo, Department of Surgery and Anatomy, Ribeirão Preto, Brazil; Ribeirão Preto Medical School, University of São Paulo, Physiotherapy Department, Ribeirão Preto, Brazil

## Introduction

Weaning from mechanical ventilation is defined as the process of release of ventilatory support and how the evaluation of this phase is conducted in the Spontaneous Breathing Test (SBT). One of the most used modes of SBT is the Continuous Positive Airway Pressure (CPAP). However, together with the mechanical ventilation modes it can be used the Automatic Tube Compensation (ATC), which compensates the resistance imposed by the endotracheal tube.

## Objectives

The main goal of this study was to compare the Oxygen Consumption (VO_2_), Carbon Dioxide Production (VCO_2_) and Energy Expenditure (EE) by Indirect Calorimetry (IC) during the SBT in CPAP with and without ATC.

## Methods

The study was a prospective randomized, controlled crossover trial that enrolled 40 patients admitted to the Intensive Care Unit of a University Hospital. Participants were randomly allocated in Group 1, in which it was started the SBT in CPAP with ATC and later in CPAP without ATC, or in Group 2, which was started the SBT in CPAP without ATC and then CPAP with ATC.

## Results

Table 1 and graph 1 summarize the differences between the treatments ATC and without ATC concerning metabolic and respiratory variables.

**Table 1:** Table 1

VARIABLES	ESTIMATED DIFFERENCES BETWEEN THE ATC AND WITHOUT ATC	p VALUE	95% CONFIDENCE INTERVAL
VO2 (Oxygen Consumption) (mL/kg.min-¹)	-1.6	0.23	[ -4.36; 1.07 ]
EE (Energy Expenditure) (kcal/day-¹)	-5.4	0.500	[ -21.67; 10.79 ]
VCO2 (Carbon Dioxide Production) (mL/kg.min-¹)	0.3	0.82	[ -2.49; 3.11 ]
RQ (Respiratory Quotient)	0.0004	0.63	[ -0.01; 0.02 ]
Peak pressure (cm H20)	2.00	0.0001	[1.39; 2.62]
Tidal volume (mL)	13.32	0.5415	[ -30.65; 57.30 ]
Respiratory rate (bpm)	0.34	0.5688	[ -0.87; 1.56 ]
P0.1 -Airway occlusion pressure 100 milliseconds after onset of inspiratory flow (cm H2O)	-0.49	0.0073	[ -0.84; -0.14 ]

## Conclusions

There were no metabolic differences evidenced by indirect calorimetry for VO_2_, EE and VCO_2_ during the SBT with and without ATC.

**Figure 1:**
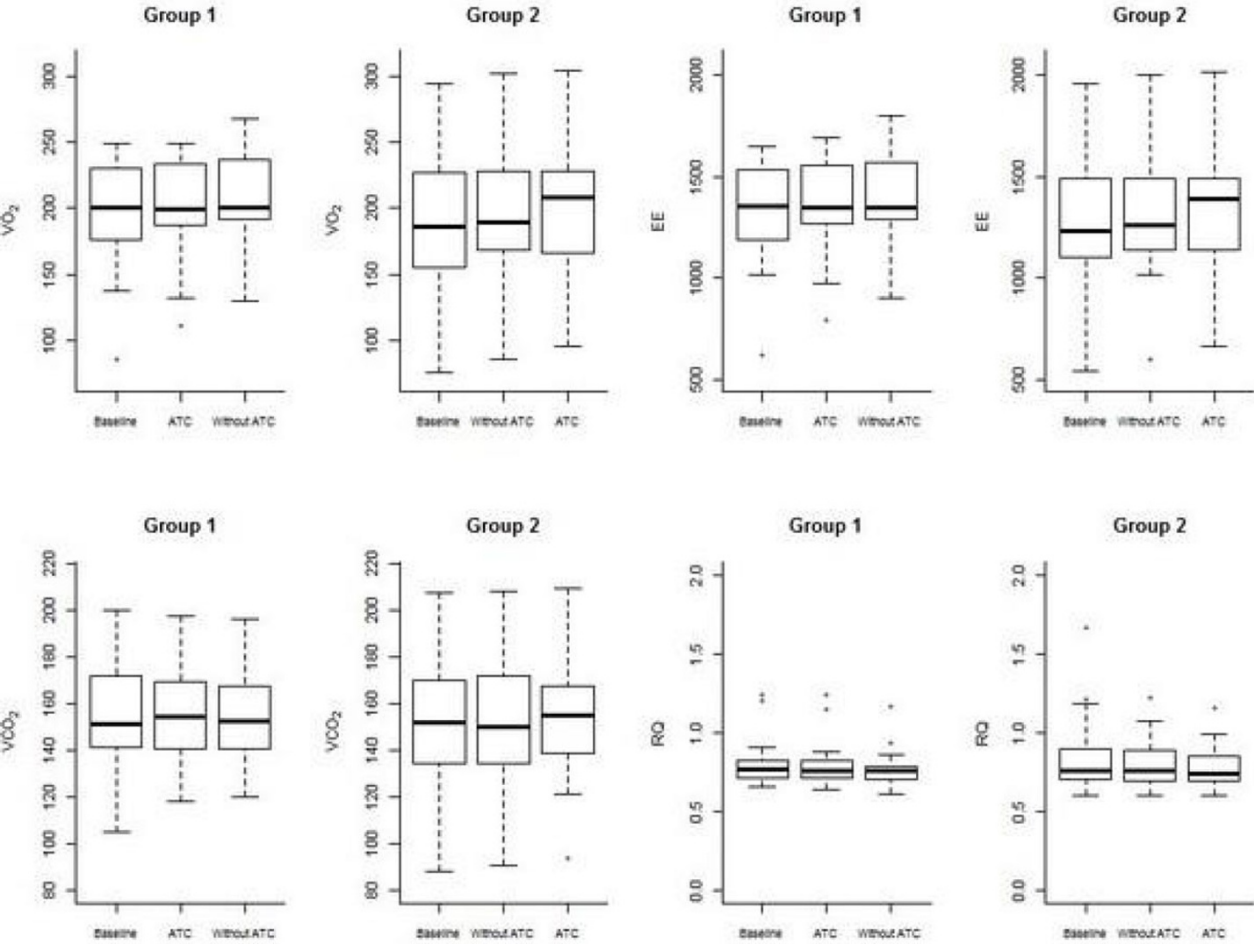
Figure 1
